# Social Media as a Sentinel for Disease Surveillance: What Does Sociodemographic Status Have to Do with It?

**DOI:** 10.1371/currents.outbreaks.cc09a42586e16dc7dd62813b7ee5d6b6

**Published:** 2016-12-07

**Authors:** Elaine O. Nsoesie, Luisa Flor, Jared Hawkins, Adyasha Maharana, Tobi Skotnes, Fatima Marinho, John S. Brownstein

**Affiliations:** Institute for Health Metrics and Evaluation, Global Health, University of Washington, Seattle, Washington, USA; Public Health Graduate Program, Escola Nacional de Saude Publica (ENSP/Fiocruz), Rio de Janeiro, Brazil; Informatics Program (BCH); Pediatrics (HMS), Boston Children's Hospital, Harvard Medical School, Boston, Massachusetts, USA; Biomedical & Health Informatics, University of Washington, Seattle, Washington, USA; Health Policy and Management, Harvard School of Public Health, Boston, Massachusetts, USA; NCDs and Health Promotion, Ministry of Health, Brasilia, Federal District, Brazil; Boston Children’s Hospital, Harvard Medical School, Boston, Massachusetts, USA

**Keywords:** Brazil, dengue, disease surveillance, infectious disease, social medi, sociodemographic status, socioeconomic factors, Twitter

## Abstract

Introduction: Data from social media have been shown to have utility in augmenting traditional approaches to public health surveillance. Quantifying the representativeness of these data is needed for making accurate public health inferences.

Methods: We applied machine-learning methods to explore spatial and temporal dengue event reporting trends on Twitter relative to confirmed cases, and quantified associations with sociodemographic factors across three Brazilian states (São Paulo, Rio de Janeiro, and Minas Gerais) at the municipality level.

Results: Education and income were positive predictors of dengue reporting on Twitter. In contrast, municipalities with a higher percentage of older adults, and males were less likely to report suspected dengue disease on Twitter. Overall, municipalities with dengue disease tweets had higher mean per capita income and lower proportion of individuals with no primary school education.

Conclusions: These observations highlight the need to understand population representation across locations, age, and racial/ethnic backgrounds in studies using social media data for public health research. Additional data is needed to assess and compare data representativeness across regions in Brazil.

## INTRODUCTION

Dengue is a mosquito-borne disease transmitted between humans by infected *Aedes* mosquitoes^1^ and is a major cause of illness and death in many tropical and subtropical regions^2^. Despite improvements in disease surveillance and investments in mosquito control programs, dengue remains a major public health threat in many countries^3^^,^ 4 ^,^ 5 ^,^ 6. Efforts at improving surveillance have explored non-traditional data sources, including, crowd-generated approaches using mobile phones and social media^7^^,^ 8, and Internet search query data^9^^,^ 10 ^,^ 11. These systems have the potential to capture mild infections not requiring medical attention, and enable the ascertainment of the probable temporal and spatial distribution of cases prior to official reports of disease.

Where available, disease reports on social media platforms also have the advantage of having geographical coordinates (latitude and longitude), enabling the probable estimation of the exact location of the case report, and potential for prompt response and vector control. In spite of this resource, in-depth analyses at fine geographical resolutions to understand temporal and spatial variation of dengue reporting using these non-traditional sources and an understanding of key sociodemographic determinants is lacking.

Using geotagged dengue disease event tweets from October 2012 to December 2014, we explore spatio-temporal dengue event reporting trends on Twitter relative to confirmed cases, and quantify key sociodemographic determinants across three Brazilian states (São Paulo, Rio de Janeiro, and Minas Gerais) at the municipality level. Brazil’s comprehensive dengue surveillance system covers over 200 million individuals^12^^,^ 13, thereby enabling a detailed assessment of this data resource.

## RESULTS

To extract major features distinguishing irrelevant and relevant (i.e. suspected dengue disease) tweets, we considered emojis, location information (state, county and micro-region), unigrams, bigrams and trigrams. We compared three machine-learning classifiers - Support Vector Machines (with linear, sigmoid, radial basis function kernels), Naïve Bayes and Maximum entropy. The accuracy of the different classifiers was evaluated using a sample of the data and a five-fold cross validation process. The Naïve Bayes classifier with a linear kernel based on a feature set combining text unigrams and emojis performed best (Table 1). The precision, recall and F1-score (a measure of accuracy) of relevant tweets were 75.20%, 80.51% and 77.52%, respectively and 90.23%, 87.34% and 88.66%, respectively for irrelevant tweets. The macro-averaged precision, recall and F1-score of the system were 82.72%, 83.93% and 82.72%. The most significant text features (Table 2) suggest that individuals are more likely to tweet of dengue if a family member or associate is ill, or to express sadness, or pain or discuss death due to dengue disease.

**Table 1:** Comparison of classifier performance

Classifier with Feature Sets	Naive Bayes (Unigrams Only)	Naive Bayes (Unigrams + Emojis)	Naive Bayes (Unigrams + Emojis + Bigrams)	Linear SVM (Unigrams + Emojis)
Accuracy	84.45	85.06	86.22	84.91
Precision	74.21	75.20	81.20	77.17
Recall	79.74	80.51	75.53	76.02
True Negative Rate	86.83	87.34	91.48	89.17

**Table 2:** The most representative unigram features

Features - 'Irrelevant' tweets	English Translation	Features - 'Sickness' tweets	English Translation
parado	stopped	irmã / irmão/ vo/ mãe/ pai/ prima / professor	sister / brother / grandfather/ mother /father / cousin / professor
mosquito	mosquito	dores / dói	pains / it hurts
ebola	ebola	:( ...	emojis
copa	cup	hospital	hospital
dando	giving	morrendo	dying
veneno	poison	repouso	rest
saúde	cheers	resultado	result

**Figure 1:**
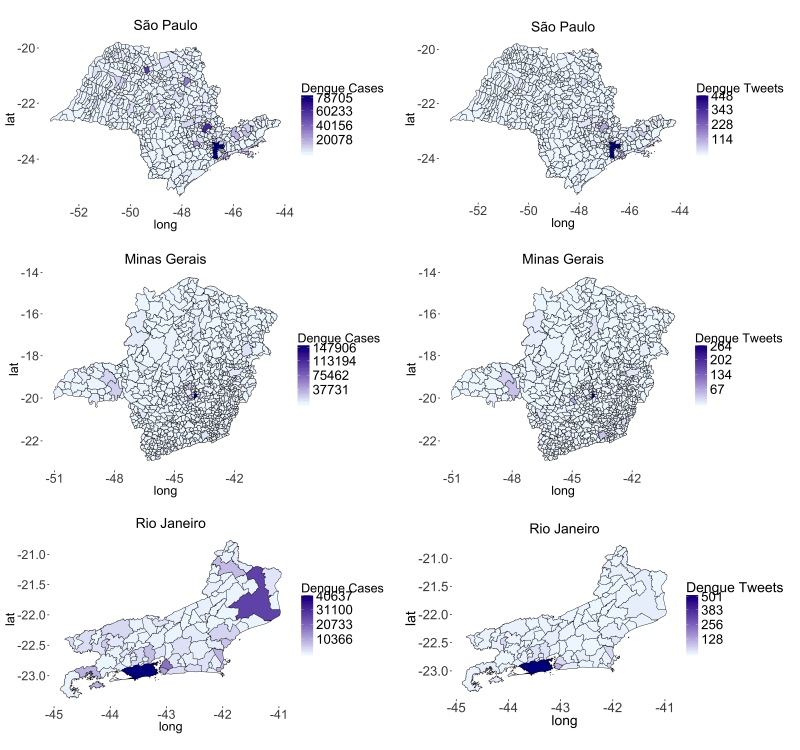
**Figure 1.** Spatial variation of case and tweet volume by municipality across the states of São Paulo, Minas Gerais, and Rio de Janeiro for 2013-2014.

Suspected dengue cases were reported on Twitter from all states in Brazil from 2013 to 2015 (Figure SI 1) and the highest volume of reports originated from São Paulo (3204 reports; 41.39%), Rio de Janeiro (1368 reports; 17.67%) and Minas Gerais (1025 reports; 13.24%). These reports were distributed across 254 (39.38%), 143 (16.78%) and 64 (69.57%) municipalities in São Paulo, Minas Gerais and Rio de Janeiro, respectively. The tweet volume was significantly much lower compared to dengue case volume and densely populated municipalities tended to have higher dengue case and tweet volume across all states (Figure 1).

**Sociodemographic Analysis**

The best logistic multivariable model to predict the occurrence of dengue tweets included population density and the percentage of individuals with higher education, older adults; defined as 60 and above, and males. A high percentage of individuals with higher education at the municipality level was positively associated (0.14, 95% CI [0.11, 0.16]) with dengue reporting on Twitter. In contrast, a higher percentage of older adults (-0.12, 95% CI [-0.18, -0.06]), and males (-0.32, 95% CI [-0.47, -0.18]) at the municipality level were negatively associated with the observation of a dengue tweet. Compared to the other variables, population density was only mildly predictive (p=0.04) of dengue disease reporting on Twitter. Additionally, a 1% increase in income was associated with a 2.89% increase in the odds of observing a dengue tweet in a municipality. These differences were more marked for municipalities in Rio de Janeiro compared to Minas Gerais.

**Figure 2:**
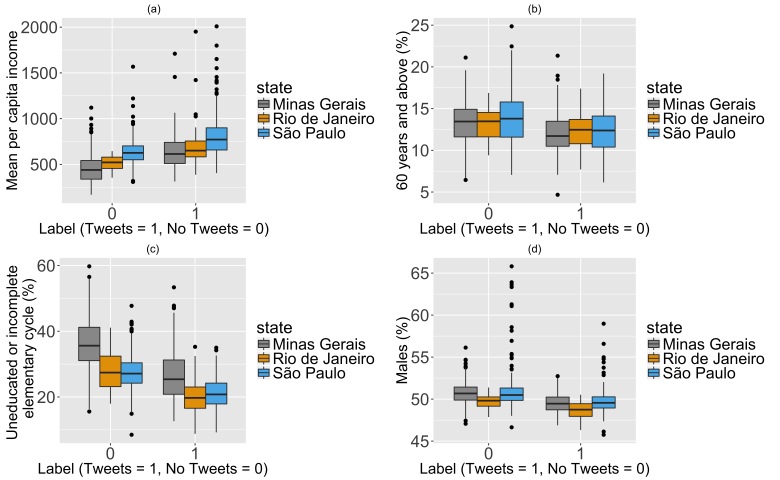
**Figure 2.** Comparison of the distribution of (a) mean per capita income; (b) percent population 60 years and older; (c) percent population without basic education; and (d) percent population identified as male between municipalities with and without tweets.

**Temporal Analysis**

We fit univariate linear regression models to state-level dengue case data, with weekly tweet volume as the independent variable for each of the three states. Despite less than 50% of municipalities accounting for the tweets for two of the three states, weekly tweet volume explained 53.65% (correlation (r) = 0.74), 85.69% (r = 0.93) and 67.98 % (r = 0.82) of the variance observed in the confirmed weekly dengue cases for the states of São Paulo, Minas Gerais and Rio de Janeiro, separately.

**Figure 3:**
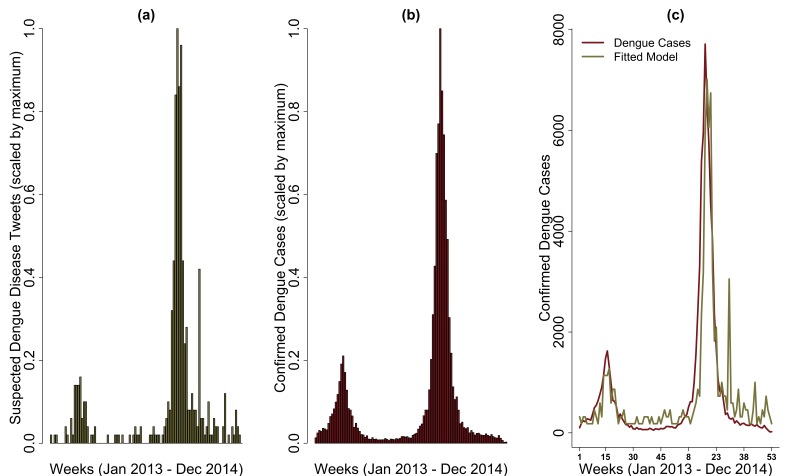
**Figure 3.** (a) and (b) are scaled weekly volume of tweets of suspected dengue disease and confirmed dengue cases for the municipality of São Paulo, São Paulo, respectively. (c) univariate linear regression model of weekly dengue cases fitted against weekly suspected dengue disease tweets.

Univariate linear regression models fit to weekly tweet volume for the municipalities of São Paulo in São Paulo, Belo Horizonte in Minas Gerais, and Rio de Janeiro in Rio de Janeiro had similar outcomes. Weekly tweet volume from the municipality of São Paulo explained 77.47% (r = 0.88) of the variance observed in the confirmed weekly case data (Figure 3(a) and (b)). Similarly, weekly tweet volume for Belo Horizonte and Rio de Janeiro separately explained 81.41 % (r = 0.90) and 56.36 % (r = 0.68) of the variance observed in the confirmed weekly case data.

Dengue cases peaked a week prior to the suspected dengue disease tweets for both São Paulo and Belo Horizonte municipalities. In contrast, the dengue tweets peaked two weeks prior to dengue cases for Rio de Janeiro, suggesting tweets could be predictive of dengue case volume. Additionally, weekly volume of tweets of suspected dengue cases captured dynamical changes in reported cases, which differed significantly across municipalities in the same state (e.g., São Paulo (Figure 3(b)) and Santos (SI 6) in the state of São Paulo). However, such associations were only observed for municipalities with a high tweet volume, suggesting that state-level aggregation of such data excludes some municipalities with confirmed dengue cases.

## DISCUSSION

Real-time reports of dengue on social media can potentially be used to augment disease response time; resulting in quicker control efforts and mitigation of disease spread. Although the majority of tweets are suspected cases, laboratory confirmed cases are also reported and real-time reports provide timely updates for situational awareness^14^, which is necessary due to weekly or monthly delays in dengue case reporting in Brazil and other endemic regions.

Inequality and low mean per capita income have been associated with dengue mortality in Brazil^15^. Furthermore, males, and people older than 69 years had a higher mortality rate from neglected tropical diseases, when compared to other populations in Brazil from 2000 to 2011^16^. Our results indicate that these populations – lower educated, males, and people older than 60 – are less likely to tweet about dengue disease. This suggests that social media might not be an adequate supplement to traditional public health surveillance for these populations.

The rapid penetration of the Internet and mobile phone technology has provided a great opportunity for improving data collection in data poor regions. However, different communities use varied forms of technology to communicate and some portions of the population (e.g., individuals with little or no basic education) might lack access and the knowledge to use certain technologies. Therefore, studies that aggregate these data across spatial and temporal scales, may only represent major cities or regions with higher education and income, thereby excluding poorer regions.

A limitation of this study is that only approximately 1 to 4% of tweets are geotagged, thereby leading to a small sample size for most municipalities. In addition, some of the suspected dengue cases are likely due to other disease etiologies and a denominator for scaling the tweet volume is unavailable. Additional data is needed to explore representativeness and differences across regions in Brazil.

Despite these limitations, significant correlations were observed between tweets and actual case reports. Two approaches for improving the utility of these data for public health surveillance are to integrate data from different sources, and develop methods to improve estimations in data poor scenarios to enable representation of poor and at-risk populations^17^. Participatory surveillance systems could be useful in supplementing these data if at-risk individuals can be convinced to participate. In addition to surveillance, these data can be used for seeding mathematical models for assessment of control strategies and real-time updates of disease occurrence reports^18^. Suspected cases in a municipality can be later confirmed as additional data become available. If combined with mobility, environmental and socioeconomic covariates, there is potential for assessing the potential spread and quantifying the impact of different intervention methods during ongoing disease epidemics, such as zika and chikungunya, that share the same vectors as dengue^19^.

Our results suggest that populations that have been shown to have a higher dengue mortality risk are also less likely to tweet about dengue. Studies aiming at augmenting dengue surveillance using these data should make careful inferences, while accounting for the caveats associated with these data resource, including the underrepresentation of specific populations.

## MATERIALS AND METHODS

**Dengue Case Data**

De-identified dengue case reports were provided by the Brazilian Ministry of Health for October 2012 to December 2014. We further aggregated the data to daily and weekly totals for each municipality and state. The cases comprised of dengue hemorrhagic fever, dengue shock syndrome and dengue fever.

**Dengue Reports from Twitter**

We extracted from Twitter - a social networking site - a subset of tweets containing the term “dengue” or hashtags with dengue (e.g., #eutenhodengue) posted between October 2012 and May, 2015, for Brazil. This was done by 1) writing a custom script in PHP to access the free Twitter Public API to collect the maximum allowed number of tweets (up to 1% total volume) with any geographical coordinates (either tweet coordinates or place coordinates), and then 2) restricting to those tweets with coordinates that were within the geographic bounding box for Brazil.

**Tweet Classification**

We developed a large manually curated sample of tweets by classifying each tweet as irrelevant, official report, or relevant (suspected dengue disease case). Two curators independently classified each tweet and tweets with curator agreement (8,000 of 10,116) were used to train a machine learning classifier and to assess human-machine agreement. A standard two step classification approach involving, pre-processing and evaluation of three machine-learning classifiers - Support Vector Machines (with linear, sigmoid, radial basis function kernels), Naïve Bayes and Maximum entropy was used.

All manually classified tweets were assigned to a training or test set. Each tweet was pre-processed and represented as a feature vector. This involved tokenization (separation of sentences into individual words), stemming and removal of stop and common words, not typically useful for classification. To extract major features distinguishing irrelevant and relevant tweets, we considered emojis, location information (state, county and micro-region), unigrams, bigrams and trigrams. The accuracy of the different classifiers was evaluated using the test data and a five-fold cross validation process. The cross validation involves randomly partitioning the data into a training and validation set prior to applying the classifiers. This process is repeated five times and the results are averaged. The best performing machine learning classifier was applied to the 14,611 unclassified tweets in the database and 2,207 tweets with curator disagreement. All tweets were reverse geo-located to extract the municipality and state of origin. Python was used for these analyses.

**Spatio-temporal Analysis**

We used the resulting dataset – manually tagged and machine classified tweets – to describe spatial and temporal trends in reporting, and evaluated key sociodemographic determinants on the reporting of dengue on Twitter using logistic regression after considering a mixed effects logistic regression model. The response variable was represented as one if reports of dengue on Twitter could be mapped to a municipality and zero otherwise. We explored different combinations of the six covariates from the Brazilian census (www2.datasus.gov.br) – sex (male or female), age (under five, five to fourteen, fifteen to thirty-nine, forty to fifty-nine, and sixty and above), race (white, brown, black, yellow, indigenous and undeclared), level of education (uneducated or incomplete elementary cycle, complete primary cycle or 2nd cycle incomplete, and 2nd cycle complete or more), mean per capita income and population density at the municipality level. Since the levels of the various variables were highly correlated, we evaluated four models with main differences in the level of the education variable and age group considered. Additionally, univariate linear regression models and Pearson correlation were used to quantify temporal association between tweets and dengue case data. The response variable was a time series of the number of confirmed dengue cases and the dependent variables was the number of relevant tweets. The model was fit for municipalities with a high volume of relevant dengue tweets. These analyses were implemented in R.

## Authors' Contributions

EON, LSF, and TS manually classified tweets. JBH, JSB, LSF, and FM provided data. AM implemented the machine learning classifier. EON drafted the manuscript. All authors read and edited the manuscript.

## Data Availability

Python code used in analyses are available on Github: https://github.com/adypooja/dengueTweets. The dengue case data is publicly available from Brazil Ministry of Health SINAN system (http://sinan.saude.gov.br/sinan).

## Competing Interest Statement

John S. Brownstein is a member of the PLOS Currents: Outbreaks review board.

## Corresponding Author

Elaine Nsoesie: onelaine@vt.edu

## Appendix

**Figure:**
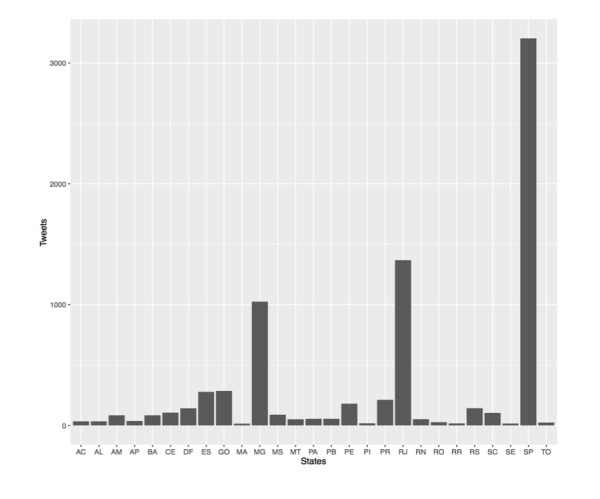
**Figure SI 1.** Number of dengue tweets from each state in Brazil. There was at least one tweet of a suspected dengue case from each of the states with the highest volume originating from São Paulo, Rio de Janeiro and Minas Gerais.

**Figure:**
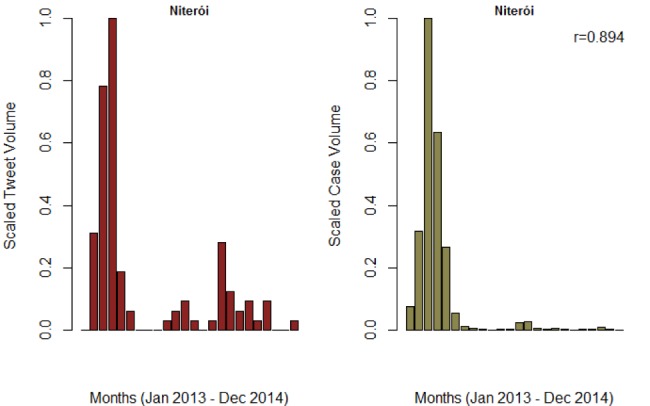
**Figure SI 2.** Trend of monthly tweet volume and confirmed cases for Niteroi municipality in Rio de Janeiro. The estimated Pearson correlation was 0.894 and 0.708 for monthly and weekly reports, respectively.

**Figure:**
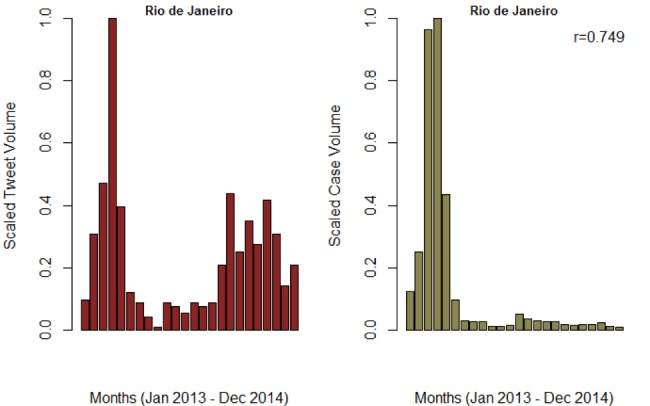
**Figure SI 3.** Trend of monthly tweet volume and confirmed cases for Rio de Janeiro municipality in Rio de Janeiro. The Pearson correlation wa3s 0.749 and 0.683 for monthly and weekly reports, respectively.

**Figure:**
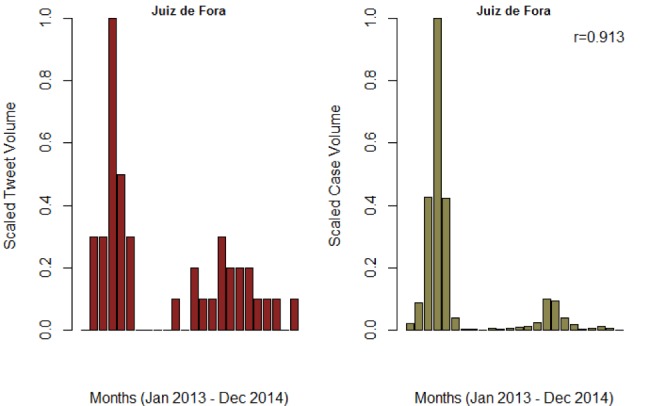
**Figure SI 4.** Trend of monthly tweet volume and confirmed cases for Juiz de Fora municipality in Minas Gerais. The Pearson correlation was 0.913 and 0.524 for monthly and weekly reports, respectively.

**Figure:**
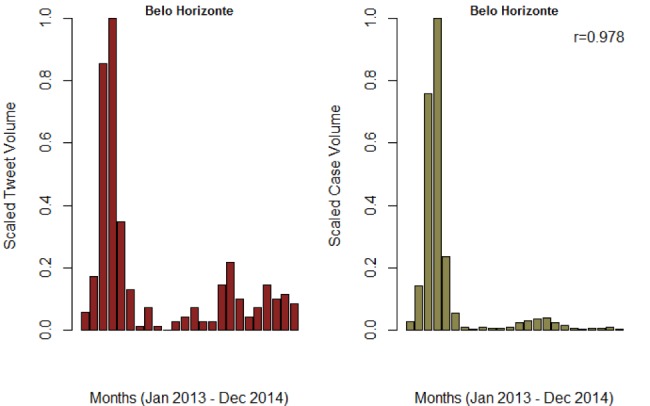
**Figure SI 5.** Trend of monthly tweet volume and confirmed cases for Belo Horizonte municipality in Minas Gerais. The Pearson correlation was 0.978 and 0.903 for monthly and weekly reports, respectively.

**Figure:**
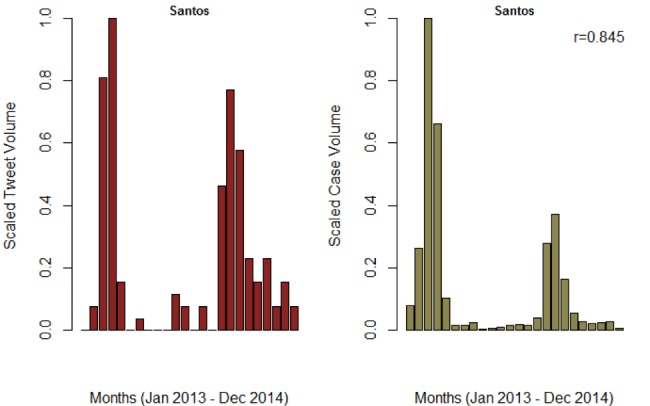
**Figure SI 6.** Trend of monthly tweet volume and confirmed cases for Santos municipality in São Paulo. The Pearson correlation was 0.845 and 0.689 for monthly and weekly reports, respectively.

## References

[1] Kraemer MU, Sinka ME, Duda KA, Mylne AQ, Shearer FM, Barker CM, Moore CG, Carvalho RG, Coelho GE, Van Bortel W, Hendrickx G, Schaffner F, Elyazar IR, Teng HJ, Brady OJ, Messina JP, Pigott DM, Scott TW, Smith DL, Wint GR, Golding N, Hay SI. The global distribution of the arbovirus vectors Aedes aegypti and Ae. albopictus. Elife. 2015 Jun 30;4:e08347. PubMed PMID:26126267. 26126267 10.7554/eLife.08347 PMC4493616

[2] Bhatt S, Gething PW, Brady OJ, Messina JP, Farlow AW, Moyes CL, Drake JM, Brownstein JS, Hoen AG, Sankoh O, Myers MF, George DB, Jaenisch T, Wint GR, Simmons CP, Scott TW, Farrar JJ, Hay SI. The global distribution and burden of dengue. Nature. 2013 Apr 25;496(7446):504-7. PubMed PMID:23563266. 23563266 10.1038/nature12060 PMC3651993

[3] Horstick O, Tozan Y, Wilder-Smith A. Reviewing dengue: still a neglected tropical disease? PLoS Negl Trop Dis. 2015 Apr 30;9(4):e0003632. PubMed PMID:25928673. 25928673 10.1371/journal.pntd.0003632 PMC4415787

[4] Murray NE, Quam MB, Wilder-Smith A. Epidemiology of dengue: past, present and future prospects. Clin Epidemiol. 2013 Aug 20;5:299-309. PubMed PMID:23990732. 23990732 10.2147/CLEP.S34440 PMC3753061

[5] Achee NL, Gould F, Perkins TA, Reiner RC Jr, Morrison AC, Ritchie SA, Gubler DJ, Teyssou R, Scott TW. A critical assessment of vector control for dengue prevention. PLoS Negl Trop Dis. 2015 May 7;9(5):e0003655. PubMed PMID:25951103. 25951103 10.1371/journal.pntd.0003655 PMC4423954

[6] Erlanger TE, Keiser J, Utzinger J. Effect of dengue vector control interventions on entomological parameters in developing countries: a systematic review and meta-analysis. Med Vet Entomol. 2008 Sep;22(3):203-21. PubMed PMID:18816269. 18816269 10.1111/j.1365-2915.2008.00740.x

[7] Wójcik OP, Brownstein JS, Chunara R, Johansson MA. Public health for the people: participatory infectious disease surveillance in the digital age. Emerg Themes Epidemiol. 2014 Jun 20;11:7. PubMed PMID:24991229. 24991229 10.1186/1742-7622-11-7 PMC4078360

[8] Gomide J, Veloso A, Meira W Jr, Almeida V, Benevenuto F, Ferraz F, et al. Dengue Surveillance Based on a Computational Model of Spatio-temporal Locality of Twitter. Proceedings of the 3rd International Web Science Conference. New York, NY, USA: ACM; 2011. pp. 3:1–3:8. doi:10.1145/2527031.2527049

[9] Chan EH, Sahai V, Conrad C, Brownstein JS. Using web search query data to monitor dengue epidemics: a new model for neglected tropical disease surveillance. PLoS Negl Trop Dis. 2011 May;5(5):e1206. PubMed PMID:21647308. 21647308 10.1371/journal.pntd.0001206 PMC3104029

[10] Gluskin RT, Johansson MA, Santillana M, Brownstein JS. Evaluation of Internet-based dengue query data: Google Dengue Trends. PLoS Negl Trop Dis. 2014 Feb 27;8(2):e2713. PubMed PMID:24587465. 24587465 10.1371/journal.pntd.0002713 PMC3937307

[11] Althouse BM, Ng YY, Cummings DA. Prediction of dengue incidence using search query surveillance. PLoS Negl Trop Dis. 2011 Aug;5(8):e1258. PubMed PMID:21829744. 21829744 10.1371/journal.pntd.0001258 PMC3149016

[12] Teixeira MG, Siqueira JB Jr, Ferreira GL, Bricks L, Joint G. Epidemiological trends of dengue disease in Brazil (2000-2010): a systematic literature search and analysis. PLoS Negl Trop Dis. 2013 Dec 19;7(12):e2520. PubMed PMID:24386496. 24386496 10.1371/journal.pntd.0002520 PMC3871634

[13] Brady OJ, Smith DL, Scott TW, Hay SI. Dengue disease outbreak definitions are implicitly variable. Epidemics. 2015 Jun;11:92-102. PubMed PMID:25979287. 25979287 10.1016/j.epidem.2015.03.002 PMC4429239

[14] Codeco C, Cruz O, Riback TI, Degener CM, Gomes MF, Villela D, et al. InfoDengue: a nowcasting system for the surveillance of dengue fever transmission. bioRxiv. 2016; doi:10.1101/046193

[15] Paixão ES, Costa Mda C, Rodrigues LC, Rasella D, Cardim LL, Brasileiro AC, Teixeira MG. Trends and factors associated with dengue mortality and fatality in Brazil. Rev Soc Bras Med Trop. 2015 Jul-Aug;48(4):399-405. PubMed PMID:26312928. 26312928 10.1590/0037-8682-0145-2015

[16] Martins-Melo FR, Ramos AN Jr, Alencar CH, Heukelbach J. Mortality from neglected tropical diseases in Brazil, 2000-2011. Bull World Health Organ. 2016 Feb 1;94(2):103-10. PubMed PMID:26908960. 26908960 10.2471/BLT.15.152363 PMC4750431

[17] Scarpino SV, Scott JG, Eggo R, Dimitrov NB, Meyers LA. Data Blindspots: High-Tech Disease Surveillance Misses the Poor. Online Journal of Public Health Informatics. 2016;8.

[18] Hay SI, George DB, Moyes CL, Brownstein JS. Big data opportunities for global infectious disease surveillance. PLoS Med. 2013;10(4):e1001413. PubMed PMID:23565065. 23565065 10.1371/journal.pmed.1001413 PMC3614504

[19] Nsoesie EO, Kraemer MU, Golding N, Pigott DM, Brady OJ, Moyes CL, Johansson MA, Gething PW, Velayudhan R, Khan K, Hay SI, Brownstein JS. Global distribution and environmental suitability for chikungunya virus, 1952 to 2015. Euro Surveill. 2016 May 19;21(20). PubMed PMID:27239817. 27239817 10.2807/1560-7917.ES.2016.21.20.30234 PMC4902126

